# Molecularly targeted co-delivery of a histone deacetylase inhibitor and paclitaxel by lipid-protein hybrid nanoparticles for synergistic combinational chemotherapy

**DOI:** 10.18632/oncotarget.14742

**Published:** 2017-01-19

**Authors:** Hima Bindu Ruttala, Thiruganesh Ramasamy, Bijay Kumar Poudal, Yongjoo Choi, Ju Yeon Choi, Jeonghwan Kim, Sae Kwang Ku, Han-Gon Choi, Chul Soon Yong, Jong Oh Kim

**Affiliations:** ^1^ College of Pharmacy, Yeungnam University, 214-1, Dae-dong, Gyeongsan, 712-749, South Korea; ^2^ College of Korean Medicine, Daegu Haany University, Gyeongsan, 712-715, South Korea; ^3^ College of Pharmacy, Institute of Pharmaceutical Science and Technology, Hanyang University, Sangnok-gu, Ansan 426-791, South Korea

**Keywords:** paclitaxel, histone deacetylase inhibitor, albumin, transferrin, lipid bilayer

## Abstract

In this study, a transferrin-anchored albumin nanoplatform with PEGylated lipid bilayers (Tf-L-APVN) was developed for the targeted co-delivery of paclitaxel and vorinostat in solid tumors. Tf-L-APVN exhibited a sequential and controlled release profile of paclitaxel and vorinostat, with an accelerated release pattern at acidic pH. At cellular levels, Tf-L-APVN significantly enhanced the synergistic effects of paclitaxel and vorinostat on the proliferation of MCF-7, MDA-MB-231, and HepG2 cancer cells. Vorinostat could significantly enhance the cytotoxic potential of paclitaxel, induce marked cell apoptosis, alter cell cycle patterns, and inhibit the migratory capacity of cancer cells. In addition, Tf-L-APVN showed prolonged circulation in the blood and maintained an effective ratio of 1:1 (for paclitaxel and vorinostat) throughout the study period. In HepG2 tumor-bearing mice, Tf-L-APVN displayed excellent antitumor efficacy and the combination of paclitaxel and vorinostat significantly inhibited the tumor growth. Taken together, dual drug-loaded Tf receptor-targeted nanomedicine holds great potential in chemotherapy of solid tumors.

## INTRODUCTION

Paclitaxel (PTX) is an important chemotherapeutic drug with a broad spectrum of activity against multiple solid tumors. PTX exhibits anticancer effects through stabilization of microtubules, by blocking the β tubulin subunit of the mitotic spindle through mitotic arrest [1–3]. The therapeutic efficacy of PTX is marred by its high toxicity, poor aqueous solubility, and poor biodistribution. Numerous therapeutic efforts have been made to overcome the limitations of PTX; however, none of the approaches tested have been quite satisfying [4–7]. With this in view, the Food and Drug Administration (FDA) approved albumin-bound PTX nanoparticles (Abraxane^®^) for treatment of various cancers [8]. Although Abraxane^®^ showed some initial positive response, it still has many potential limitations including hypersensitivity reactions and hematological side effects. The most important limitation is its low colloidal stability, which results in a poor pharmacokinetic pattern in the systemic circulation [9]. Active drug PTX dissociates from albumin within 3–4 h of intravenous administration and possesses a C_max_ value of 13 μg/mL after 3 h, which drops to 1 μg/mL at 4 h with a single dose of 260 mg/m^2^ in human patients [10]. Consequently, efforts have been made to develop novel delivery systems, which can improve the colloidal stability of the nanoparticle in the systemic circulation and sustain PTX blood levels.

To this end, we propose that encapsulation of an albumin-PTX nanoparticle (NP) within a biocompatible lipid bilayer could, in principle, improve the biodistribution and the pharmacokinetic profile of the albumin conjugate vis-à-vis principle drug, PTX. This kind of lipid bilayer-supported albumin conjugate with surface PEGylation is expected to prolong the blood circulation that will aid the permeation of tumor capillaries and extravasation into tumor fenestrations via the enhanced permeation and retention (EPR) effect [11, 12]. The therapeutic efficacy of passively targeted NP is far from optimal [13, 14]. In this context, present study explores the feasibility of tumor targeting using transferrin (Tf) as a targeting ligand. The Tf receptor (TfR) is overexpressed in cancer cells (100 fold) compared to normal cells [15–17]. Therefore, Tf could be utilized as a suitable targeting ligand to deliver therapeutics to TfR-overexpressing cancer cells.

Recently, combinational regimens have been developed to decrease the adverse effects associated with high doses of a single drug and at the same time augment the therapeutic potential of individual drugs by synergy [18, 19]. Meta-analyses of multicenter clinical trials indicate that drug combination regimens exhibit synergistic tumor response rate, higher than that of a single agent [20, 21]. Vorinostat (VOR), a histone deacetylase inhibitor (HDACi) plays a crucial role in the epigenetic transcriptional regulation through stabilization of histone-DNA interactions, which induce cell cycle arrest and apoptosis of cancer cells [22]. The co-loading of VOR and PTX (from APVN, an albumin PTX/VOR-loaded nanoparticle) in a well-designed nanocarrier (L-APVN) could effectively modify the pharmacokinetics and toxicity profiles of cocktail combinations, control the release of drugs, and maintain synergistic drug ratios for maximum therapeutic benefits [23, 24].

**Figure 1:**
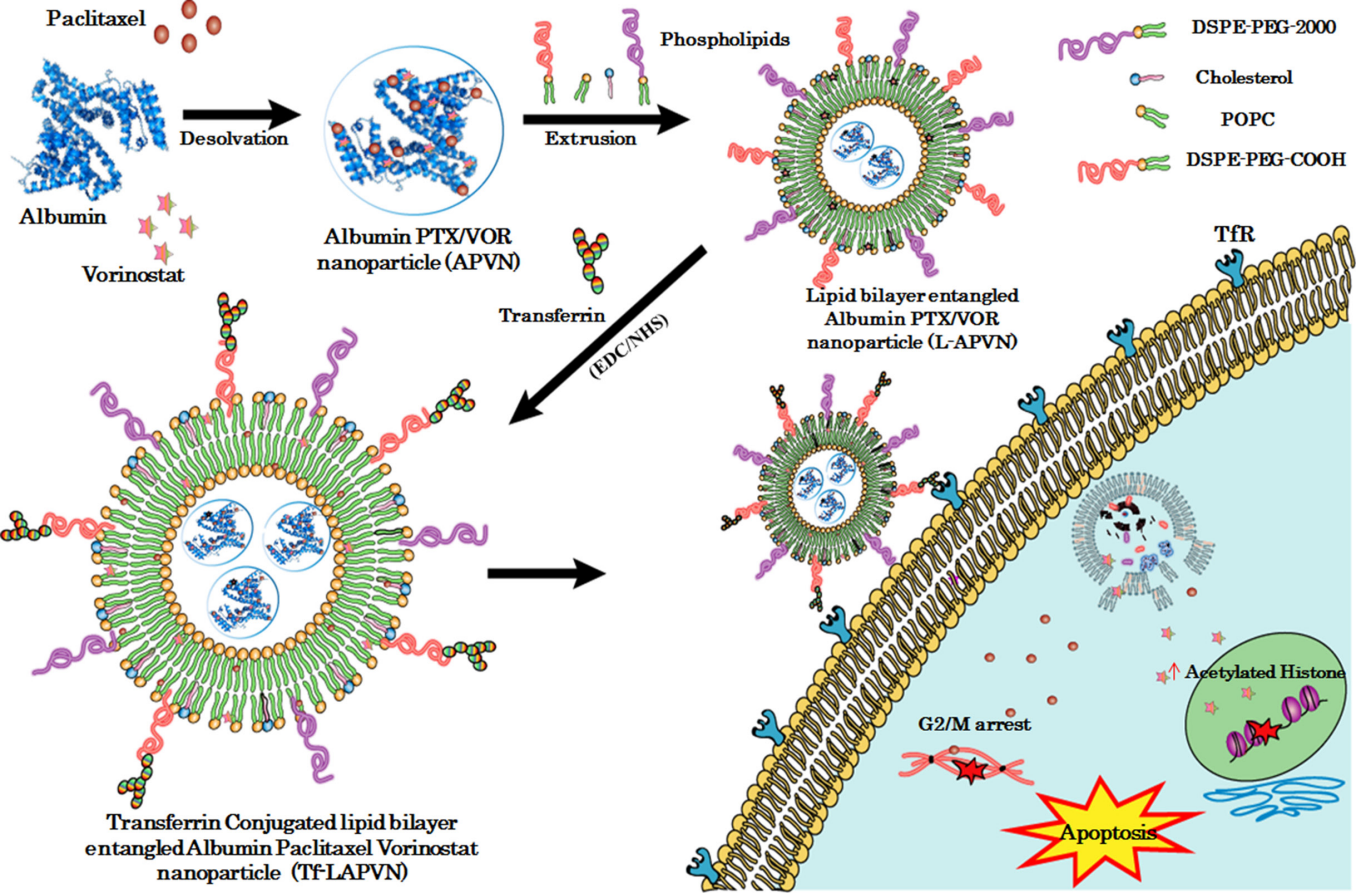
Schematic illustration of preparation of ligand-directed albumin conjugate-supported lipid bilayer for combinational co-delivery of paclitaxel and vorinostat The PTX/VOR (PV) loaded albumin conjugate (APVN) was prepared and supported with PEGylated lipid bilayer (L-APVN). The lipid bilayer-supported albumin nanocarrier was covalently conjugated with transferrin ligand (Tf-L-APVN) to design an actively targeted delivery vehicle.

We contemplated the design of lipid bilayer encapsulation of APVN, followed by the loading of second drug (VOR) and Tf conjugation (Figure 1). The main objectives of the present study were to (a) improve the colloidal stability and systemic performance of albumin NP; (b) investigate the synergistic activity of combinational drugs; (c) contemporaneously target solid tumors with high specificity. For this purpose, we have developed a custom-designed procedure in which thin lipid-film layer was applied to the round-bottomed glass surface, overlaid with PTX/VOR-loaded albumin NP, and subsequently surface conjugated with Tf. Following drug synergy investigations at an *in vitro* level, we have demonstrated the therapeutic synergistic outcome in a xenograft tumor model.

## RESULTS

### Physicochemical characterization of transferrin-conjugated lipid bilayer supported APVNs (Tf-L-APVN)

In this study, we have formulated APVN with a mean diameter of ~ 130 nm with a slightly negative charge. As expected, assembly of lipid bilayer on APVN significantly increased the particle diameter to ~ 195 nm and showed a ζ-potential of -14.7 mV (Supplementary Table 1). The final particle size after Tf conjugation was observed to be ~ 230 nm with good dispersity index (polydispersity index (PDI) ~ 0.203). The amine functional groups of Tf were covalently conjugated with the carboxylic groups of the distal terminal PEG (DSPE-PEG) present on the external nanoparticle surface. A coupling efficiency of ~ 78% was observed suggesting the success of the conjugation technique.

**Figure 2:**
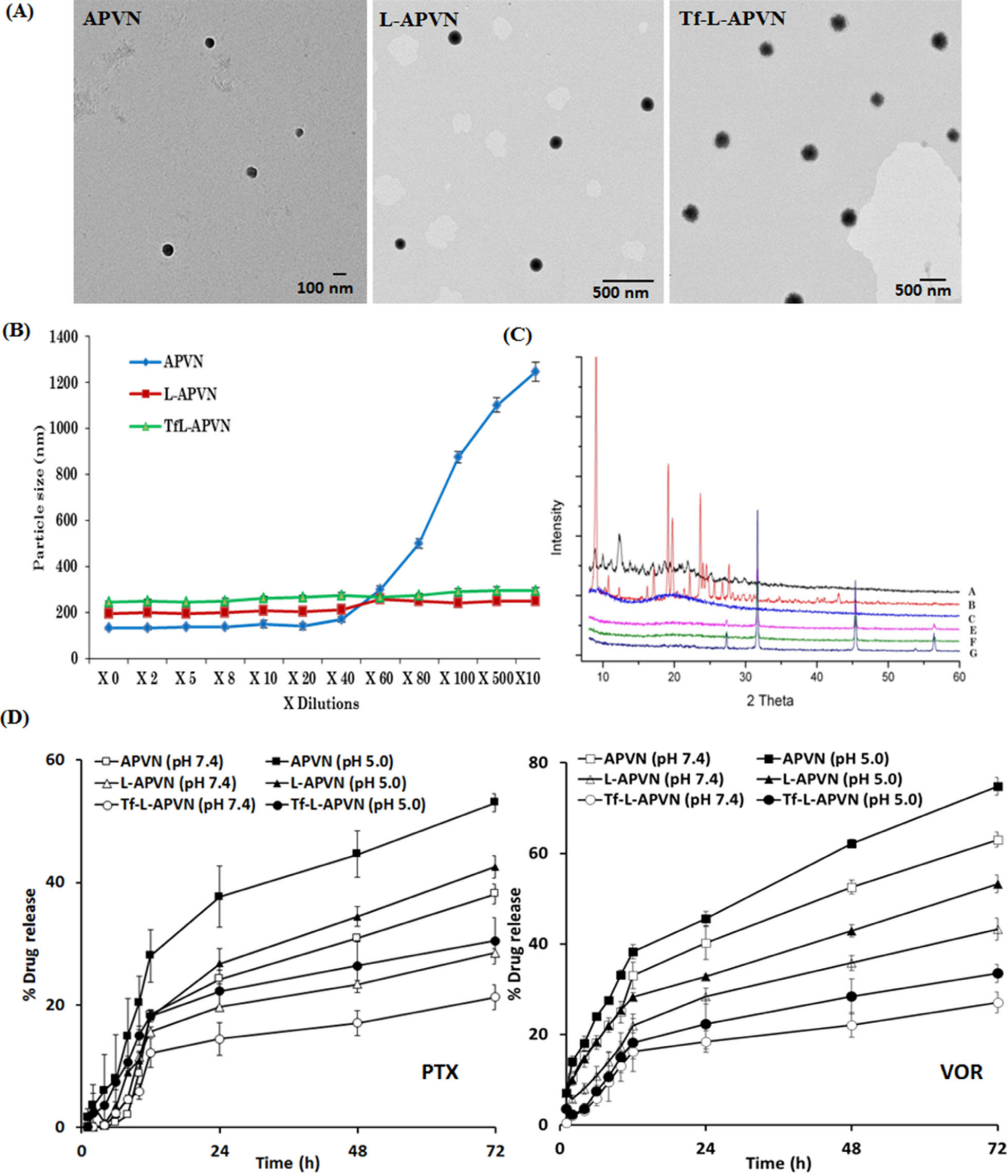
Physicochemical characterization of Tf-L-APVN (**A**) TEM images of APVN, L-APVN, and Tf-L-APVN. (**B**) Colloidal stability of APVN, L-APVN, and Tf-L-APVN upon multi-fold dilutions with buffer. (**C**) X-ray diffraction patterns of free PTX (a), free VOR (b), BSA (c), APVN (e), L-APVN (f), and Tf-L-APVN (g). (**D**) *In vitro* release profile of PTX and VOR from APVN, L-APVN, and Tf-L-APVN in PBS and ABS. The release was carried out at 37°C and data are shown as mean ± SD (*n* = 3).

TEM revealed the presence of distinct, discrete, and spherical particles, which are uniformly dispersed in the copper grid (Figure 2A). Consistent with the DLS analysis, particles were nanosized and showed incremental addition upon Tf conjugation. The colloidal stability of Tf-L-APVN in systemic circulation is one of the foremost requirements for cancer targeting applications. The colloidal stability of nanoparticles was evaluated by DLS (Figure 2B). As expected, particle size of APVNs immediately increased upon dilution by a factor of 20 due to the aggregation or disassembly of albumin carriers. In contrast, L-APVN and Tf-L-APVN maintained the same particle size even when diluted in phosphate-buffered saline (PBS) by a factor of 100, indicating their excellent colloidal stability. The presence of the protective lipid bilayer coating prevented the dissociation of albumin NPs and improved their stability parameters in agreement with previous reports [25].

### Solid-state characterization

The solid-state characterization was performed by various techniques including DSC, XRD, and FTIR. The DSC thermograms of PTX, VOR, BSA, blank liposome, APVN, L-APVN, and Tf-L-APVN are presented in Supplementary Figure 1A. The lack of these endothermic transition peaks in the formulations clearly indicates the presence of drugs in the amorphous molecular form. The XRD patterns of all the components are presented in Figure 2C. The free drugs exhibited numerous sharp and intense peaks at various scattering angles (2 θ) of 10.81, 11.92, 12.90, 15.26, 16.81, 21.56, 25.089, and 42.16° (PTX) and 16.3, 17.2, 19.2, 19.8, 22.2, and 23.7° (VOR) implying their high crystalline nature. A complete lack of these diffraction peaks in drug-loaded formulations indicates the presence of drugs in the amorphous forms [26]. FTIR analysis was performed to evaluate the chemical interactions of drugs with protein or liposomal components. The spectra of various formulations are shown in Supplementary Figure 1B. The PTX and VOR exhibited characteristic peaks at 2965 cm^−1^ (= C–H), 1707 cm^−1^ (C = O group), 1641 cm^−1^ (C–C stretch), 1370 cm^−1^ (CH_3_ bending), 1248 cm^−1^ (C–N stretch), 1072 cm^−1^ (C–O stretch), and 709 cm^−1^ (C–H off the plane). Since these peaks were also present unchanged in the spectra of liposomal formulations, likely no chemical interactions occurred between the drugs and the carrier components. Moreover, linkage between –COOH group of PEG and –NH_2_ group of transferrin was confirmed by the amide (–CO–NH–) stretching peak at 1634 cm^−1^. The signals at 1655 cm^−1^, 1537 cm^−1^, and 1396 cm^−1^ indicate amide-I, II, and III bonds in albumin and transferrin, respectively.

### *In vitro* release kinetics

The rate and kinetics of drug release from different nanoparticulate systems were evaluated in PBS (pH 7.4) and ABS (pH 5.0) conditions (Figure 2D). Neither of the drugs exhibited a burst release phenomenon. A biphasic release profile was observed for both drugs. The release profiles of Tf-L-APVNs and other formulations were significantly higher at acidic pH (5.0) than at physiological pH (7.4). Importantly, a distinct and sequential release pattern was observed for PTX and VOR, with the latter releasing faster. For example, ~ 43% of VOR released compared to ~ 28% of PTX from L-APVN at 72 h. Similarly, ~ 27% and ~ 21% of VOR and PTX were respectively released from Tf-L-APVN after 72 h incubation in PBS.

### *In vitro* cytotoxicity assay and synergy analysis

**Figure 3:**
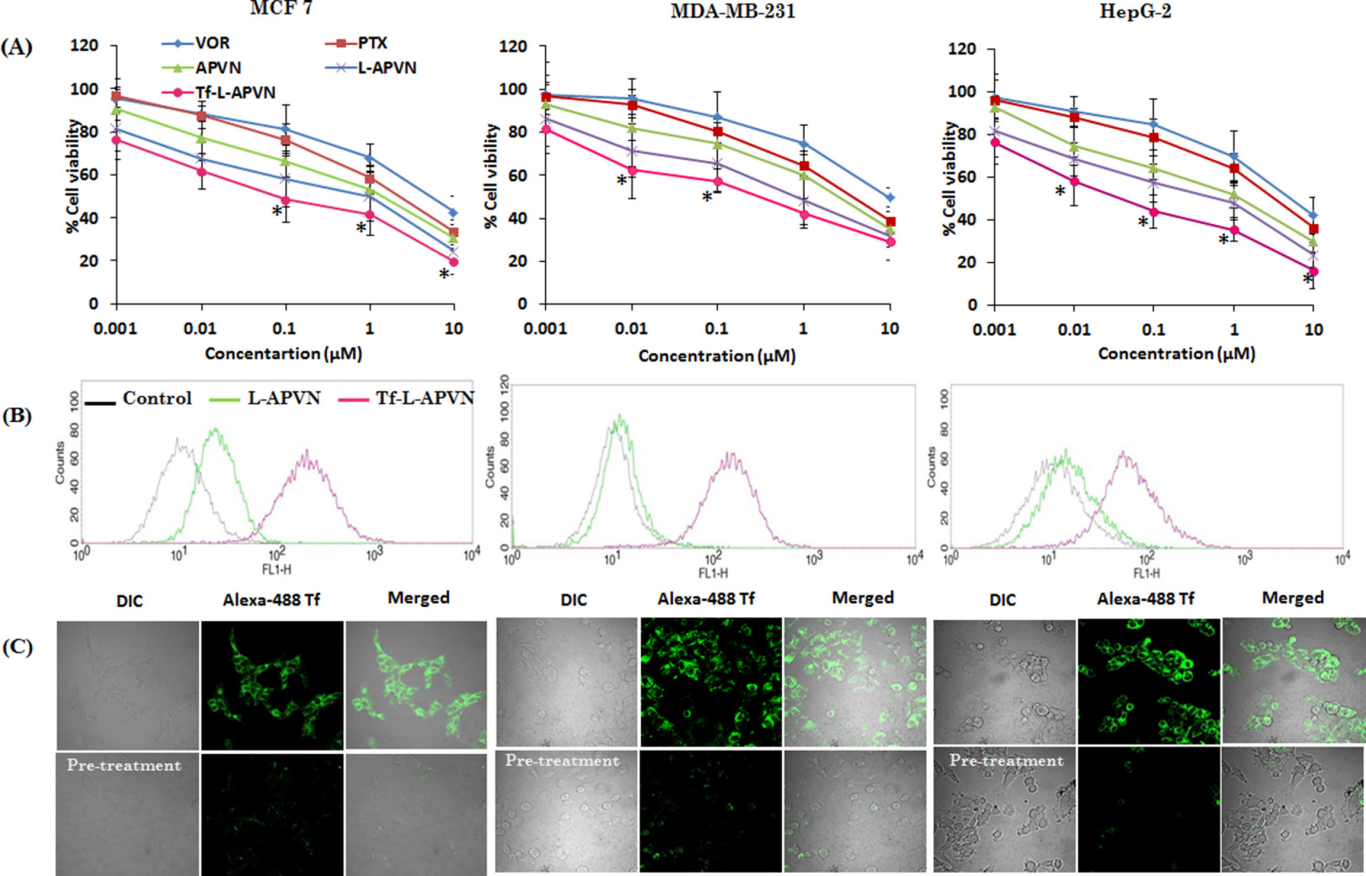
(**A**) *In vitro* dose-dependent cytotoxic effect of different formulations viz. free PTX, VOR, APVN, L-APVN, and Tf-L-APVN in a dose-dependent manner in cancer cells (the synergistic combination ratio 1:1 PTX:VOR was used). (**B**) The qualitative and quantitative internalization efficiency of targeted (Tf-L-APVN) and non-targeted (L-APVN) nanoparticles in cancer cells were studied by flow cytometry. Scale bar = 100 μm. (**C**) Cellular uptake analysis of Tf-L-APVN in MCF-7, MDA-MB-231, and HepG2 cancer cells using confocal laser scanning microscope (CLSM). The CLSM analysis was performed in the absence and presence of excess free Tf to observe the specificity in cancer cells. *p < 0.05.

The cytotoxic potential of all formulations was tested in MCF-7, MDA-MB-231, and HepG2 cancer cells. At first, cytotoxicity of blank nanoparticles was tested in these cancer cells. As shown, cell viability remains more than 85% when treated with different concentrations of blank NP (0.1–100 μg/ml) regardless of cancer cells tested, indicating the non-toxic nature of the drug delivery vehicle (Supplementary Figure 2A). Various ratiometric combinations of PTX and VOR (5:1, 2:1, 1:1, 1:2, and 1:5) were studied to investigate the synergistic cytotoxicity of the drugs in MCF-7, MDA-MB-231, and HepG2 cancer cells (Supplementary Figure 2). As expected, PTX/VOR-based combinational regimen significantly enhanced the cytotoxicity in all cancer cells, however, the level of anticancer effect varied according to the ratio of the two drugs and the nature of cancer cells. To be specific, PTX/VOR combinations at a weight ratio of 2:1, 1:1, and 1:2 were more effective in reducing cell viability compared to other ratios. PTX: VOR at weight ratios of 1:1 and 2:1 showed high synergistic activity (CI: 0.25–0.70) while 5:1 and 1:2 ratio showed relatively low synergistic activity (CI: 0.7–0.9). Based on the results, it could be expected that encapsulating combinational drugs in nanoparticulate systems will further increase the therapeutic efficacy in cancers. Following free PTX and VOR, APVN (1:1), L-APVN (1:1), and Tf-L-APVN (1:1) were tested in MCF-7, MDA-MB-231, and HepG2 cancer cells. As hypothesized, Tf-conjugated liposomal nanoformulations showed significantly remarkable anticancer effects in all cancer cells tested (Figure 3A).

### Intracellular uptake and targeting potential of Tf-L-APVN

In this study, the subcellular distribution was first evaluated by flow cytometry (Figure 3B). As shown, the mean fluorescent intensity (MFI) of Tf-L-APVNs was significantly greater (8–10 fold) than that of L-APVNs in all cancer cells (Supplementary Figure 3A). Interestingly, Tf-L-APVN showed a time-dependent cellular uptake rather than short-term uptake (Supplementary Figure 3B and 3C). MFI of Tf-L-APVNs was significantly greater (8–10 fold) than that of L-APVNs in all cancer cells. To further validate the receptor-mediated endocytic uptake, competitive uptake study was performed by Tf pre-treatment in all cancer cells. Figure 3C clearly demonstrates the inhibition of uptake of Tf-L-APVN in cells pretreated with native Tf, suggesting the key role of transferrin receptor-mediated endocytosis of the nanoparticulate system. These findings indicate that the abundant Tf receptors present in MCF-7, MDA-MB-231, and HepG2 cancer cells could be utilized to effectively target cancer cells and augment the therapeutic efficacy of the encapsulated drugs.

### Cell apoptosis and cell cycle analysis

**Figure 4:**
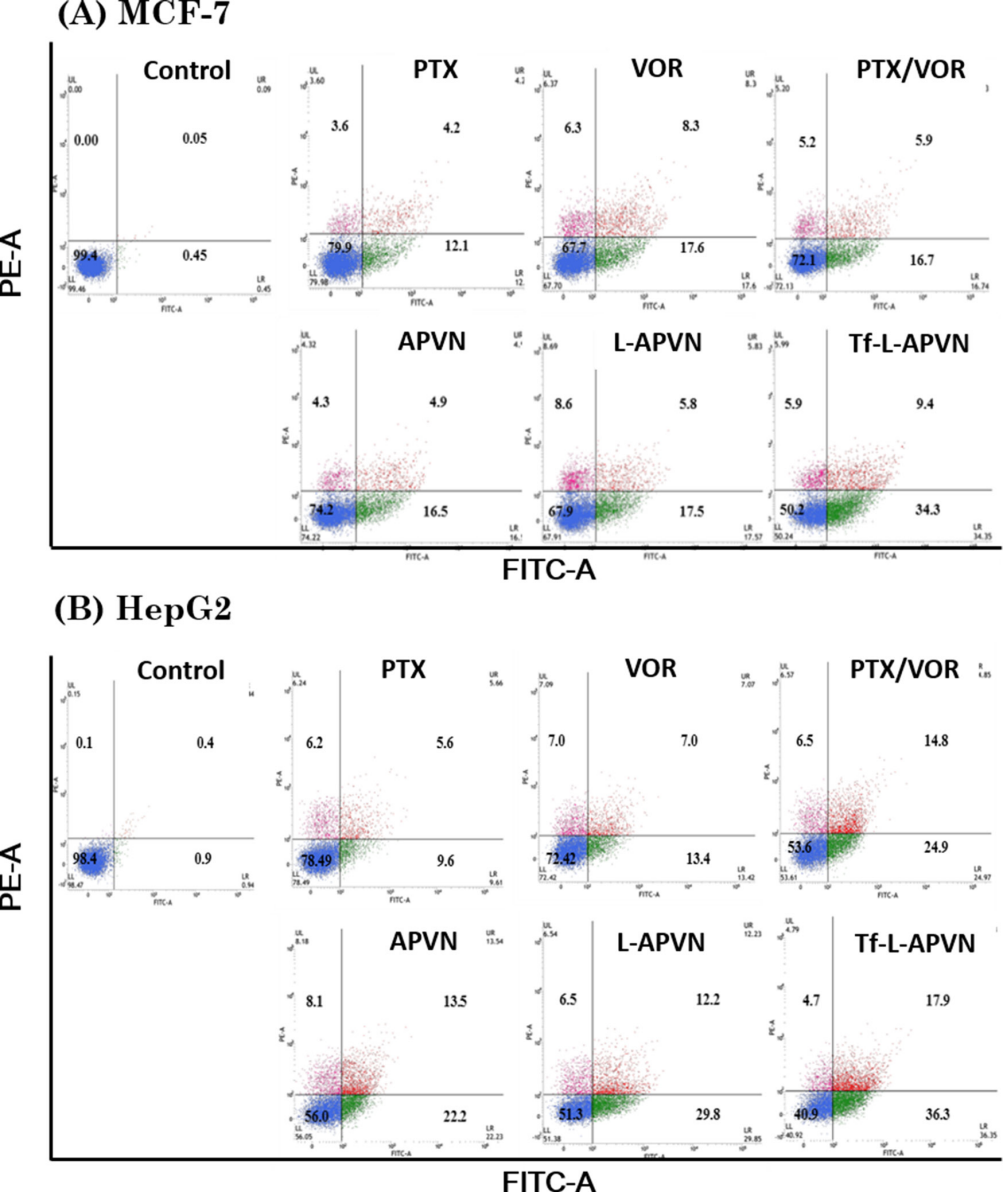
Apoptotic analysis of MCF-7 and HepG2 cells after treatment with free drugs, free drug cocktail, and combination-loaded formulations at 1 μg/mL For apoptosis assay, the cells were stained with Annexin V-FITC/PI and analyzed by flow cytometer.

Annexin V/PI assay was performed to quantify the cells undergoing apoptosis and necrosis. As shown in Figure 4, early apoptosis (annexin-V+ and PI-) and late apoptosis (annexin-V+ and PI+) were the major mechanisms of cell death caused by all the formulations. The percentage of annexin V-FITC- and PI-positive cells significantly increased when exposed to combinational nanoparticles, compared to single drugs. The free PTX or VOR did not induce appreciable apoptosis of cancer cells whereas dual drug-loaded targeted therapeutic system (Tf-L-APVN) exhibited significantly higher apoptotic effect, as evidenced by ~ 45%, ~ 30%, and ~ 58% of apoptotic cells in MCF-7, MDA-MB-231 (Supplementary Figure 4) and HepG2 cancer cells.

**Figure 5:**
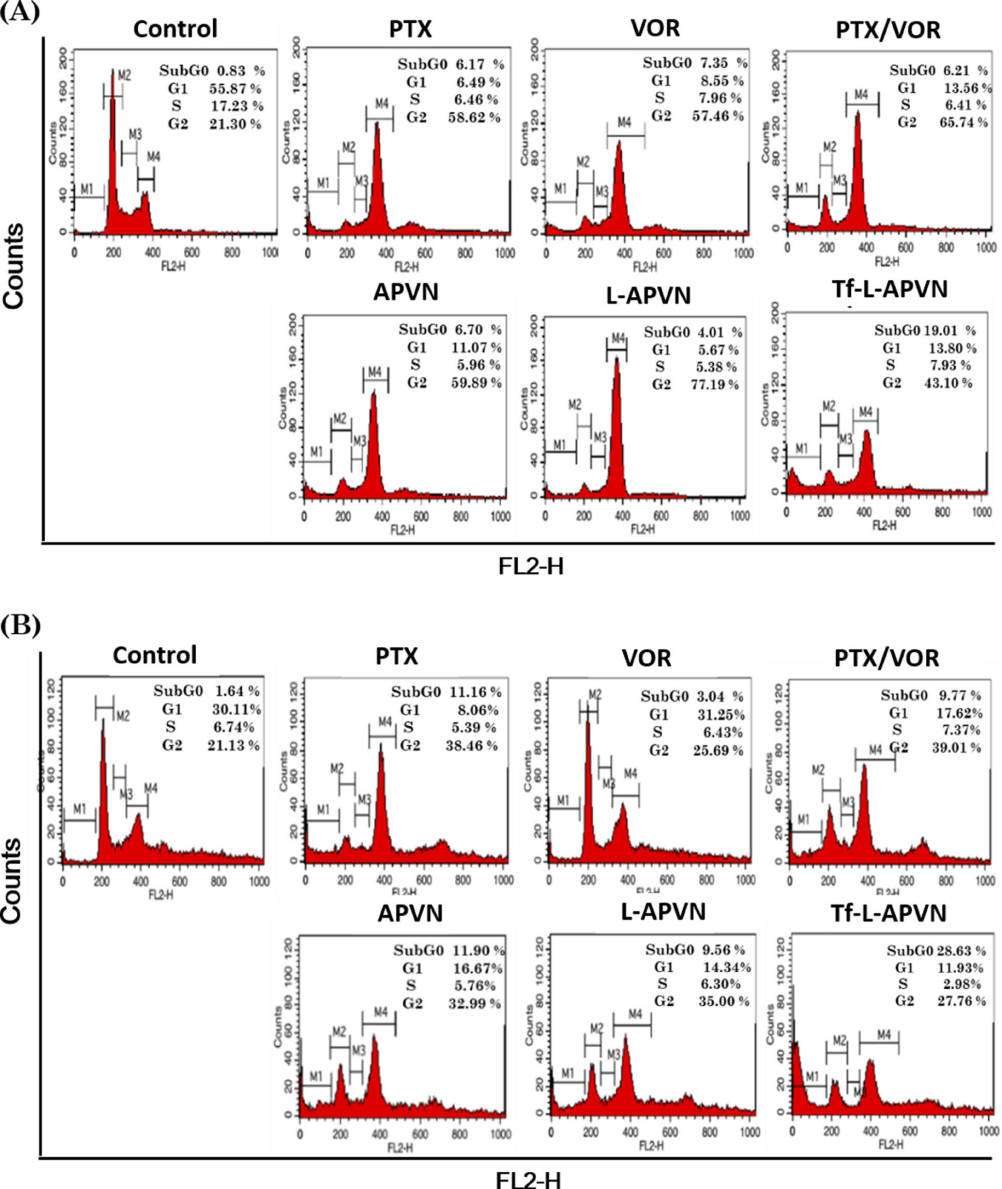
Cell cycle analysis after treatment with different formulations (1 μg/mL) in (**A**) MCF-7 and (**B**) HepG2 cells after 24 h incubation. A representative set of data from three independent experiments is shown. Cells were harvested and cell cycle phases were analyzed by staining with PI (DNA-binding dye) and RNAse using flow cytometry. G0/G1, G2/M, and S indicate the cell phase, and sub-G0/G1 refers to the proportion of apoptotic cells.

Cell cycle distribution patterns were investigated to evaluate the intracellular effects preceding the cytotoxic effect of various formulations (Figure 5). As expected, combination of PTX and VOR remarkably increased the proportion of cells in G2/M phase of cell cycle in MCF-7 and HepG 2 cancer cells. A significant increase in percentage of apoptotic cells (measured as sub-G0 content) could be detected in cell populations (~ 19–25%) treated with Tf-L-APVN, compared to other groups.

### Cell migration and regulation of intrinsic apoptotic signalling pathways

**Figure 6:**
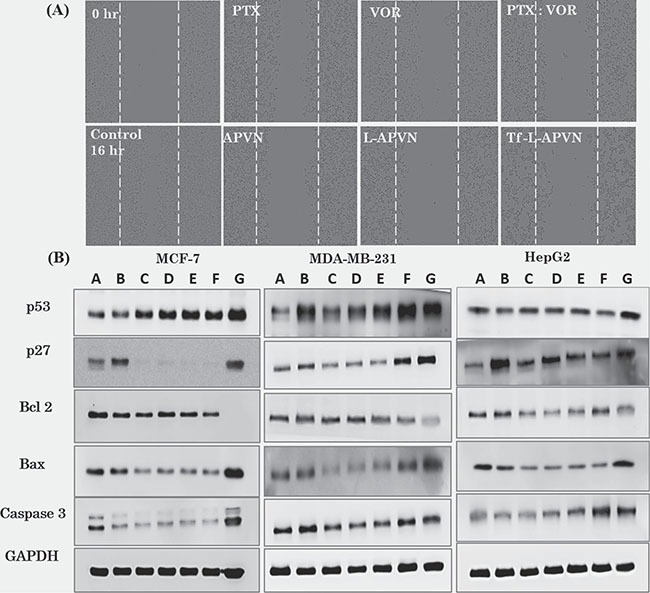
(**A**) Effect of individual formulations on the migration of MCF-7 cells. Migration was observed at 0 h and 16 h and each value was presented as the mean ± SD (*n* = 4). (**B**) Western blot analysis of BAX, p53, and GADPH expression in the three cancer cell lines. The cells were treated with various formulations at 1 μg/mL (total drug concentration) for 24 h. A: Control; B: free PTX; C: free VOR; D: PTX/VOR; E: APVN; F: L-APVN; G: Tf-L-APVN.

The wound healing test was used to determine the migratory potential of cancer cells. Figure 6A and Supplementary Figure 5 show time-dependent closure of the wounds according to the potency of the individual formulations. Free PTX and VOR inhibited the migration of MCF-7 cancer cells; however, combination regimen of PTX/VOR was more effective. Consistently, Tf-L-APVN showed significantly higher inhibitory potential compared to other formulations. The migration capacity of cells treated with Tf-L-APVN was reduced 2-fold compared to that of either PTX or VOR-treated cells indicating that the combination of these two drugs had significant synergistic effects on reducing tumor progression.

Consistent with the cytotoxicity and apoptosis analysis, Tf-L-APVN induced a marked expression of cell cycle protein p27, and p53 in MCF-7, MDA-MB-231, and HepG2 cancer cells (Figure 6B). We further monitored the change in the expression of pro-apoptotic markers (Bax and c-caspase-3) and the anti-apoptotic marker Bcl-2. Results clearly show that the Tf-L-APVN remarkably induced the expression of pro-apoptotic markers and markedly decrease the expression of anti-apoptotic markers.

### Hemolytic analysis

The safety profiles of blank NPs, free drugs and drug-loaded formulations were evaluated by a hemolysis assay (Supplementary Figure 6). As shown, free PTX (~ 25%) and free VOR (~ 40%) induced severe hemolysis of RBC, further increased by the combination cocktail (~ 50%). As expected, drug-induced hemolysis was significantly reduced when the drugs were encapsulated in nanocarriers. L-APVNs and Tf-L-APVNs in particular showed a remarkable hemoprotective property.

### Pharmacokinetic study

**Figure 7:**
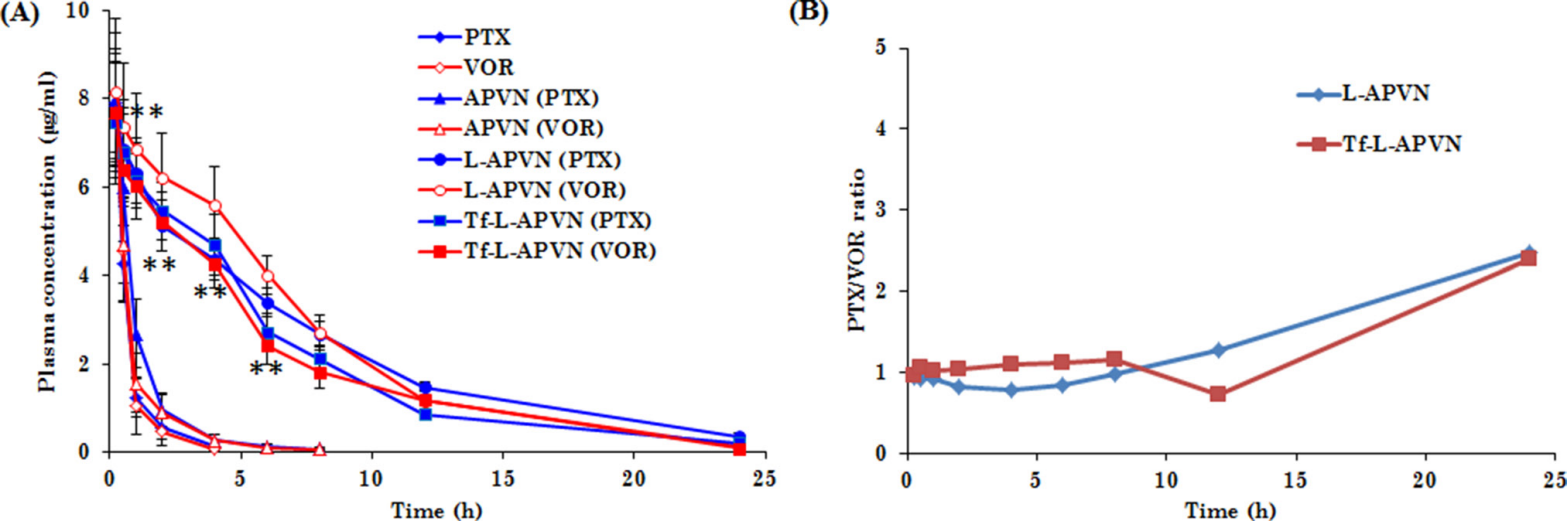
(**A**) Plasma concentration–time profiles for intravenously administered free drug cocktail (PTX/VOR), APVN, L-APVN, and Tf-L-APVN formulations in rats. The formulations were administered at a fixed dose of 5 mg/kg. (**B**) The ratio of PTX and VOR in plasma after intravenous administration. **p < 0.01 as compared with free drugs.

The plasma concentration-time profile of PTX and VOR after intravenous administration of formulations is presented in Figure 7. The poor colloidal stability of albumin conjugates resulted in the early elimination of both the drugs from systemic circulation. The pharmacokinetic parameters were analyzed by using the non-compartmental model (WinNonlin software). The terminal half-life of drugs in native free form and in albumin conjugates were in the range of 0.5–1 h, whereas terminal half-life of drugs was increased 5-fold when they were administered as L-APVNs and Tf-L-APVNs formulations (5 ~ 6 h). Similarly, PEGylated nanocarriers reduced the elimination rate constant (K*el*) of drugs 8–10 fold compared to free form. Consistently, L-APVNs and Tf-L-APVNs posted an 8–10 fold increase in the overall AUC value of PTX and VOR, indicating that nanoparticles significantly improved the blood circulation potential of encapsulated drugs (*p* < 0.01) (Table 1).

**Table 1:** Pharmacokinetic parameters of PTX and VOR after intravenous administration of a free PTX/ VOR cocktail, APVN, L-APVN, and Tf-L-APVN to rats

Parameters	Free drug cocktail		APVN		L-APVN		Tf-L-APVN	
	PTX	VOR	PTX	VOR	PTX	VOR	PTX	VOR
K_el_ (h^-1^)	1.06 ± 1.38	1.20 ± 2.47	0.62 ± 0.21	0.58 ± 1.41	0.12 ± 0.10	0.16 ± 0.87	0.15 ± 0.14^*^	0.18 ± 0.12^*^
t_1/2_ (h)	0.64 ± 0.59	0.57 ± 3.51	1.11 ± 1.21	1.18 ± 0.38	5.48 ± 1.12	4.10 ± 0.11	4.53 ± 0.87^*^	3.83 ± 0.48^*^
AUC_all_(h.μg/ml)	7.26 ± 2.54	7.09 ± 0.24	9.81 ± 2.58	8.59 ± 1.52	55.52 ± 0.52	57.91 ± 0.42	47.55 ± 1.54^*^	46.54 ± 2.41^*^
AUC_∞_(h.μg/ml)	7.36 ± 1.02	7.14 ± 0.59	9.92 ± 1.60	8.70 ± 0.78	58.36 ± 0.37	58.76 ± 0.15	48.95 ± 2.56^*^	47.03 ± 0.15^*^
Cl	13.6 ± 0.87	13.9 ± 0.12	10.1 ± 0.41	11.5 ± 1.81	1.71 ± 2.48	1.70 ± 1.48	2.04 ± 3.80^*^	2.12 ± 1.54^*^
AUMC	4.34 ± 0.64	3.8 ± 1.47	10.5 ± 0.21	9.17 ± 0.42	355.52 ± 1.94	316.14 ± 0.81	257.49 ± 1.42^*^	254.51 ± 0.10^*^
MRT	0.59 ± 0.13	0.53 ± 4.71	1.06 ± 0.34	1.06 ± 2.87	6.40 ± 0.73	5.45 ± 2.15	5.41 ± 0.48*	5.46 ± 2.32*

Data expressed as mean ± SD (*n* = 4)

*p < 0.05, **p < 0.01 for differences between Tf-L-APVN / PTX-VOR (cocktail).

### *In vivo* antitumor efficacy of combination therapeutics

**Figure 8:**
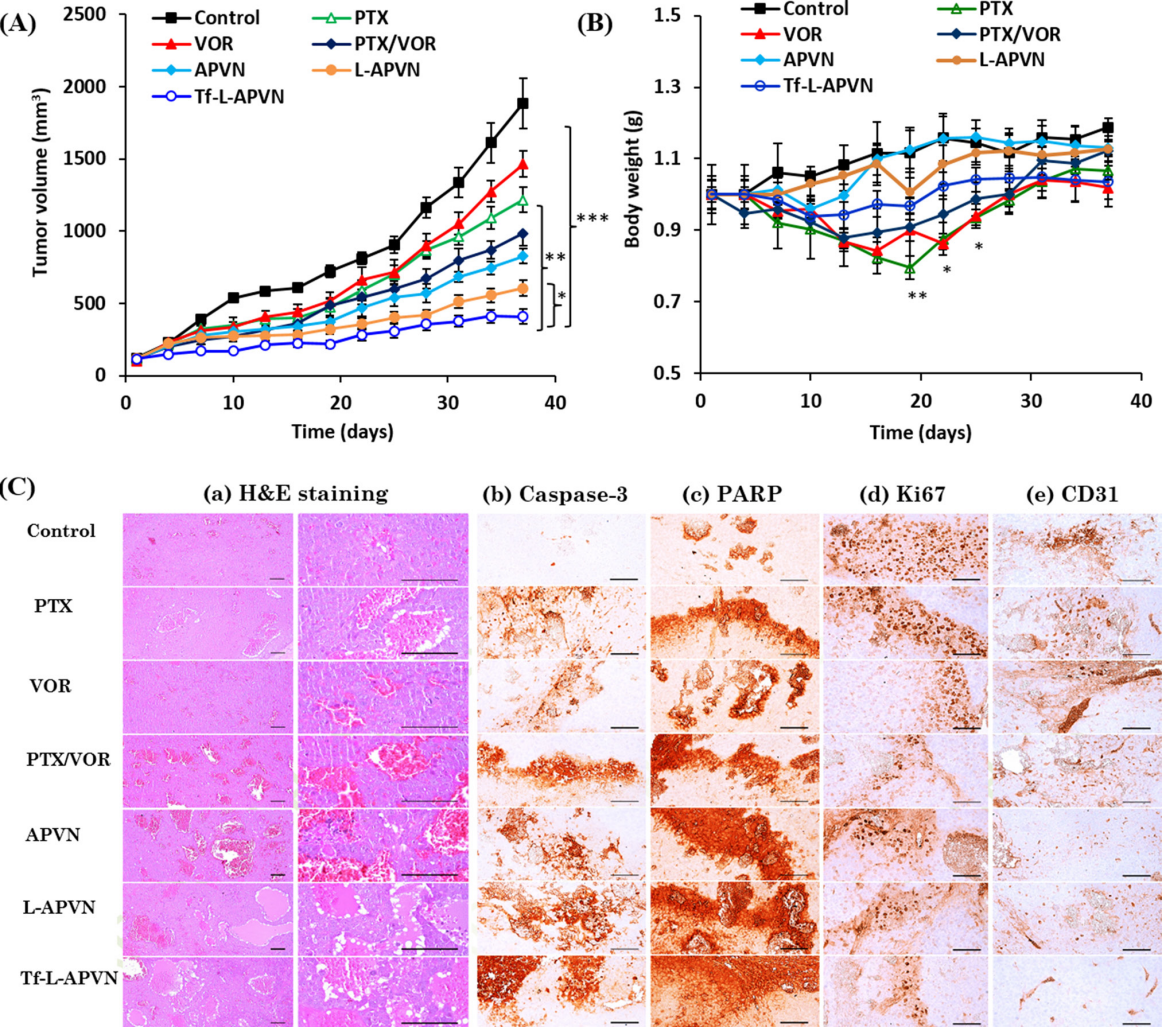
*In vivo* antitumor efficacy of combinational nanoparticles (**A**) Changes in tumor volume and (**B**) body weight in nude mice bearing HepG2 xenografts after treatment with different formulations. The formulations were administered via the tail vein at a fixed dose of 5 mg/kg on days 1, 4, 7, and 10. Data are presented as the mean ± SE (*n* = 7). (**C**) Histological and immunohistochemical analysis following different treatments. (a) H&E staining, (b) caspase-3, (c) PARP, (d) Ki67, and (e) CD31. Caspase-3 and PARP, markers for apoptosis; CD-31 and Ki-67, markers for angiogenesis; scale bars = 120 μm. *p < 0.05, **p < 0.001, ***p < 0.0001.

We have further evaluated the synergistic anticancer effect of nanoformulations in HepG2 tumor-bearing xenograft mice (Figure 8A). All formulations were administered at a fixed dose of 5 mg/kg via intravenous injections. Compared to control, free PTX and VOR slightly inhibited tumor growth, indicating that single drug treatment will not be effective in combating tumors. Although the cocktail combination and APVNs delayed the tumor growth in comparison to both free PTX and free VOR, they could not induce complete regression of tumors due to the poor distribution of drugs in the systemic circulation [27]. In contrast, Tf-L-APVNs displayed excellent antitumor efficacy compared to other groups, demonstrating that the combination treatment of dual drugs in nanocarriers could produce a synergistic antitumor effect. Animal body weight changes were used as a marker of the toxicity of anti-tumor therapeutic agents in tumor-bearing mice (Figure 8B). Compared to control, free drugs as well as cocktail combinations significantly decreased the body weight, indicating that PTX and VOR, both commonly used chemotherapeutic drugs in clinic, had great systemic toxicity. In contrast, encapsulation of dual drugs in nanocarriers obviously reduced the declining body weight index implying the excellent safety profile of the formulations.

The micrographs of H&E stained tissue sections are presented in Figure 8C. Consistent with antitumor efficacy, Tf-L-APVNs significantly decreased the tumor cell volume and induced remarkable changes in tumor microstructures, such as apoptotic condensations and fragmentation of cancer cells (Supplementary Table 2). The mice treated with PTX, VOR, PTX/VOR, APVNs, L-APVNs, and Tf-L-APVNs showed 30.34%, 26.31%, 48.16%, 43.93%, 58.52%, and 64.54% of caspase-3-immunolabeled cells, respectively, while untreated mice showed 4.26%. Similar observations were made for PARP, which showed the highest expression in animals treated with Tf-L-APVNs. The number of CD31+ and Ki-67+ cancer cells was significantly lower in the Tf-L-APVN-treated group compared to other groups.

## DISCUSSION

Albumin-based nanocarrier was developed to improve the therapeutic efficacy of PTX in solid tumors. However, this carrier suffers from poor colloidal stability resulting in rapid elimination of the drug from systemic circulation. Upon intravenous administration, albumin conjugates become unstable in a large volume of blood and release the encapsulated PTX, which is immediately removed from systemic circulation, limiting the possibility of exchange of PTX between external and body albumin. Our objectives were to improve the stability of albumin conjugates after systemic administration using a protective lipid bilayer coating, control the release of the drug in the blood circulation, and limit unwanted toxicity. These characteristics would make our therapeutic approach advantageous compared to Abraxane. With a dual objective, to improve the systemic performance of the albumin nanocarrier and to induce a synergistic combinational therapeutic effect, the present study involves developing a lipid bilayer-supported PTX and VOR-bound albumin NP (L-APVN).

The APVN was prepared by desolvation technique in which PTX attaches near site I interface of the subdomains IIA and IIIA and the cleft between domains I and III with its C13 sidechain, while VOR binds to hydrophobic cavities present in subdomains IIA and IIIA of albumin via hydrogen bonding [24]. We have covalently conjugated Tf to the surface of lipid nanoparticles (Tf-L-APVN) via EDC/NHS chemistry. In this study, Tf-L-APVN with a mean diameter of ~230 nm with good dispersity index (PDI ~ 0.203) was observed. The entrapment efficiency of drugs was determined using HPLC. The Tf-L-APVN showed a high entrapment efficiency of ~ 100% for PTX and ~ 77% for VOR. The difference in the entrapment efficiency of the two drugs was attributed to the difference in partition coefficients that might result in the competition between drugs to load in the carrier.

Consistent with our hypothesis, the presence of the protective lipid bilayer effectively controlled the release of PTX and VOR into the media. In particular, the release rates of both drugs were significantly reduced after the Tf conjugation to the lipid bilayer (Tf-L-APVN). The difference observed in the release patterns of PTX and VOR might be attributed to the disparity of the hydrophobic nature of drugs. One of the main objectives of the present study was to stabilize APVN using a lipid bilayer and to control and achieve a sustained release of the drugs. As expected, the release of the drugs was remarkably reduced after lipid bilayer encapsulation. Conjugation of Tf on the surface of NP further slowed the release of drugs, possibly owing to the high molecular mass of Tf and formation of a dense Tf layer around the nanoparticle [25, 28].

Both anticancer drugs exhibited a typical dose-dependent cytotoxic effect on sensitive (MCF 7 and HepG2) and resistant (MDA-MB-231) cancer cells. IC_50_ values were lower in sensitive cancer cells than in resistant cells, which might be due to the overexpression of p-gp efflux transporters. As expected, PTX/VOR-based combinational regimen significantly enhanced the cytotoxicity in all cancer cells, however, the level of anticancer effect varied according to the ratio of the two drugs and the nature of cancer cells. The TfR is overexpressed in proliferating tumor cells because there is a higher need for iron in these cells, as iron is a cofactor in DNA synthesis [29, 30]. Using Tf-targeted NP, we observed a sharp decrease in the IC_50_ values of individual drugs compared to non-targeted NPs. Following free PTX and VOR, APVN (1:1), L-APVN (1:1), and Tf-L-APVN (1:1) were tested in all cancer cells. As hypothesized, Tf-conjugated liposomal nanoformulations exhibited remarkable anticancer effects in all cancer cell lines.

Anticancer therapies work by inducing apoptosis in cancer cells without damaging the surrounding normal cells. The appreciable apoptotic activity of Tf-L-APVN was mainly attributed to the higher accumulation of nanoparticles in the cells via Tf-receptor mediated endocytosis uptake and the sequential release of drugs in the intracellular environment that resulted in a synergistic therapeutic effect [25, 28, 30]. In general, cell cycle distribution following DNA damage is controlled by checkpoints. PTX is known to bind to the tubulin dimer within microtubules, and therefore exhibited a G2/M phase arrest of cells, which was closely associated with the cell growth inhibition. As expected, Tf-L-APVN exhibited a remarkable cell cycle arrest. The untreated cells were largely found in the G0/G1 phase, while combinational NPs induced a significantly higher G2/M phase arrest and appreciable amount of cells in the sub-G0 phase [31]. The migration of cancer cells plays an important role in tumor progression and proliferation. The migration capacity of cells treated with Tf-L-APVN was reduced 2-fold compared to that of either PTX or VOR treated cells indicating that the combination of these two drugs had significant synergistic effects on tumor progression.

Cellular signaling pathways were investigated to demonstrate the molecular mechanism behind the anticancer effect. Tf-L-APVN induced a marked expression of cell cycle protein p27, and p53 in cancer cells. PTX stabilizes microtubules leading to G2/M phase cell cycle arrest (via a Raf-1 dependent pathway) and p27 and p53 protein induction. Based on the literature, we expected that VOR potentiates the PTX cytotoxicity by promoting microtubule stabilization through increase of tubulin acetylation, which disrupts the alignment of chromosomes during mitosis and leads to apoptosis [32, 33]. Results clearly show that the Tf-L-APVN remarkably induced the expression of pro-apoptotic markers and markedly decrease the expression of anti-apoptotic markers. The decreased expression of Bcl-2 protein in the present study might result in the formation of pores in the mitochondrial membranes, leading to proteolytic processing and activation of the pro-caspase-9 that initiates the downstream apoptosis cascade [34, 35].

To demonstrate the fact that nanoparticulate encapsulation of anticancer drugs will reduce toxic effect, we have performed the haemolytic assay. As expected, L-APVNs and Tf-L-APVNs in particular showed a remarkable hemoprotective property. The excellent hemocompatibility profile of blank NPs indicates their suitability for systemic applications [36]. The chemotherapeutic drugs such as PTX and VOR could transport into the RBC membrane and increase the osmotic pressure leading to the rupture of the cell membrane and Hb release. The presence of a protective layer around the chemotherapeutic drugs would make entering the RBCs impossible, leading to reduced side effects and rendering the system safer and more hemocompatible [37].

Pharmacokinetic study revealed that nanoparticulate system improved the circulatory ability of free drugs. Consistent with our hypothesis, lack of colloidal stability of albumin conjugates (APVNs) resulted in poor systemic performance for both drugs. In contrast, L-APVNs and Tf-L-APVNs significantly prolonged the blood circulation for PTX and VOR, maintaining therapeutic levels of drugs throughout the study period. It is noteworthy that the nanoformulations maintained an effective weight ratio of 1:1 for PTX and VOR throughout the study period, a ratio for which maximum synergistic anticancer activity was observed in the cancer cells studied. The drugs encapsulated in L-APVNs and Tf-L-APVNs exhibited a slow decline in the concentrations found in the systemic circulation. Many inferences can be drawn from these experiments. Albumin conjugates (APVNs) disassembled in the systemic environment whereas lipid bilayer coating improved the *in vivo* performance of drugs; second, antifouling property of PEG improved the circulatory profile of the delivery vehicle; third, both drugs released in a sustained and ratiometric manner (1:1) which could further improve the therapeutic efficacy [27, 38, 39].

*In vivo* anticancer efficacy study revealed that Tf-L-APVNs could effectively control the tumor progression demonstrating that the combination treatment of dual drugs in nanocarriers could produce a synergistic antitumor effect. The tumor volume of Tf-L-APVN treated mice was 4.6-fold, 3-fold, 3.6-fold and 2.4-fold smaller compared to tumor volume of mice treated with control, free PTX, free VOR, and cocktail PTX/VOR, respectively. Furthermore, the PTX formulated in the non-targeted L-APVNs and targeted Tf-L-APVNs showed significant (P < 0.05) decrease in tumor size indicating the therapeutic advantage of Tf conjugation, and of Tf as a targeting ligand. We believe that the excellent antitumor effect of Tf-L-APVN can be attributed to the tumor targeting potential of Tf that could efficiently deliver the nanoparticles and release the drug in the weakly acidic tumor microenvironment. In addition, prolonged blood circulation, pH-responsiveness and size of nanoscaled carriers, and ratiometric and synergistic effects of dual drugs all contributed to the prominent tumor regression effect of Tf-L-APVNs.

In summary, we have successfully developed a transferrin-anchored robust nanoplatform for the targeted co-delivery of PTX and VOR, in order to achieve maximum synergistic effect in solid tumors. At cellular levels, Tf-L-APVN significantly enhanced synergistic effects of PTX and VOR on the proliferation of MCF-7, MDA-MB-231, and HepG2 cancer cells. VOR could significantly enhance the cytotoxic potential of PTX, induce marked cell apoptosis, change cell cycle patterns, and inhibit the migratory capacity of sensitive and resistant cancer cells. In addition, L-APVNs and Tf-L-APVNs demonstrated prolonged circulation in the blood and maintained an effective weight ratio of 1:1 (for PTX and VOR) throughout the study period. In HepG2 tumor-bearing mice, Tf-L-APVN displayed excellent antitumor efficacy and significantly inhibited tumor growth. Taken together, these results indicate that PTX/VOR-combination drug-based TfR-targeted nanomedicine holds great potential in cancer chemotherapy of solid tumors.

## MATERIALS AND METHODS

### Materials

Vorinostat was purchased from LC Laboratories (MA, USA). Paclitaxel was obtained from Shaanxi Top Pharm Chemical Co. Ltd (Xi’an, China). Bovine serum albumin (BSA; MW = 66 000 kDa), holo-transferin (Tf), and 3-(4,5-dimethylthiazol-2-yl)-2,5-diphenyltetrazolium bromide (MTT) were purchased from Sigma–Aldrich (St. Louis, MO, USA). 1-Palmitoyl-2-oleoyl-sn-glycero-3-phosphocholine (POPC), 1,2-distearoyl-sn-glycero-3 phosphoethanolamine-N-[carboxy(polyethylene-glycol)- 2000] (DSPE-PEG2000), and cholesterol were purchased from Avanti Polar Lipids (Alabaster, AL, USA). Tf-Alexa488 and Lysotracker red were purchased from Invitrogen/Molecular Probes, Inc. (Eugene, OR). All other chemicals were of reagent grade and were used as supplied.

### Preparation of the APVNs, L-APVNs and Tf-L-APVNs

The paclitaxel (PTX) and vorinostat (VOR)-entangled albumin nanoparticles (APVNs) were prepared by desolvation [46, 47]. Briefly, PTX and VOR (250 μg/mL each) in ethanol were added drop by drop to 1 mL (1 mg/mL) of albumin solution maintained at pH 9. The albumin-drug mixture was probe sonicated for 15 min and kept aside. Separately, a thin film of the lipid mixture was prepared by adding POPC (2.6 μM), cholesterol (5.17 μM), DSPE-PEG2000 (0.36 μM) and DSPE-PEG-COOH (0.7 μM) and removing the chloroform using a rotary evaporator. Later, the thin film was hydrated with the APVN solution for 3 h with the final lipid concentration of 10 mM. The albumin-lipid suspension was extruded 11 times through a stack of two polycarbonate membranes with 200 nm pores using an Avanti Mini Extruder (Avanti Polar Lipids, Alabaster, AL, USA) resulting in the encapsulation of APVN in the lipid bilayer (L-APVN). Afterwards, transferrin (Tf) was conjugated to L-APVNs (Tf-L-APVNs). The amine functionalities of holo-transferrin (Tf) were used to conjugate DSPE-PEG-COOH by EDC/NHS-based carbodiimide chemistry. Briefly, to stirred 1 mL of the lipid formulation equivalent amounts of EDC and NHS (0.25 M) were added and allowed to incubate for 15 min at room temperature. Next, 125 μg of T_f_ in PBS (pH 7.4) was added and the mixture was incubated overnight. The unbound protein was separated from liposomes by ultracentrifugation at 12000 rpm for 30 min. The coupling efficiency was further determined by BCA protein assay and was calculated as μg Tf/μmol PL (Phopholipid).

### Morphological characterization

The nanoparticle morphology was examined using a transmission electron microscope (TEM; H7600, Hitachi, Tokyo, Japan) at an accelerating voltage of 100 kV. A drop of the NP dispersion was placed onto a copper grid coated with a carbon film, negatively stained with phosphotungstic acid (2%, w/v), and viewed under TEM. Atomic Force Microscopy (AFM) was also performed using Nanoscope IIIa scanning probe microscope (Digital Instruments, Murray Hill, NJ, USA).

### Physicochemical characterization

The hydrodynamic size, polydispersity index (PDI), and ζ-potential were analyzed by dynamic light scattering (Nano ZS 90, ZetaSizer; Malvern Instruments, Malvern, UK). The NPs were suitably diluted with distilled water and measured at 25°C. The X-ray diffraction (XRD) patterns of the samples were recorded using a vertical goniometer and X-ray diffractometer (X’Pert PRO MPD diffractometer, Almelo, The Netherlands) measuring Ni-filtered CuK-radiation (voltage, 40 kV; current, 30 mA) scattered in the crystalline regions of the sample.

### Drug encapsulation efficiency

The encapsulation efficiency and drug loading of the NP formulations were determined using Agilent 1200 series high-performance liquid chromatography (HPLC) after ultrafiltration using an Amicon® centrifugal filter device (molecular weight cutoff 10 000 Da; EMD Millipore, Billerica, MA, USA). The supernatant was injected onto a C_18_ column (Inertsil^®^ ODS3: 0.5 μm, 15 cm × 0.46 cm, GL Sciences Inc., Japan) using a gradient mobile phase comprised of acetonitrile and water with 0.1% formic acid (35:65 (VOR) and 60:40 (PTX), v/v) mobile phase with a flow rate of 1.0 mL/min (Supplementary Figure 1C). The eluent was analyzed using a UV-Vis detector at 227 nm for PTX and 241 nm for VOR, respectively.

### *In vitro* release

The release of PTX and VOR from APVNs, L-APVNs and Tf-L-APVNs was evaluated in phosphate-buffered saline (PBS, pH 7.4, 0.14 M NaCl), and acetate-buffered saline (ABS, pH 5.0, 0.14 M NaCl) using membrane tubing (Spectra/Por^®^; 3500 Da cutoff, CA, USA). 1% Tween 80 were added to increase the drug solubility in a medium. The dialysis was performed at 37 °C, at 100 rpm. The samples were collected at predetermined times and replaced with equal amounts of fresh buffer. The concentrations of PTX and VOR present in the dialysate were quantified by HPLC using Agilent HPLC as described above.

### Cell culture

The MCF-7, MDA-MB-231 and HepG2 cells (all obtained from the American Type Culture Collection, Manassas, VA, USA) were grown in Dulbecco's modified Eagle's medium (DMEM) containing 10% fetal bovine serum and 1% penicillin/streptomycin at 37°C in a humidified atmosphere containing 5% CO_2_.

### Cytotoxicity assay and synergy analysis

The cytotoxicity potential and synergy analysis of VOR and PTX was tested in MCF 7, MDA-MB-231 and HepG2 cancer cells. Cells (1 × 10^5^) were seeded in 96-well plates and incubated overnight at 37 °C. The next day, the cells were exposed to various concentrations of PTX and VOR (individually). Also, cells were co-treated with the combination of drugs at a weight ratio of 5:1, 2:1, 1:1, 1:2 and 1:5 (PTX:VOR). Furthermore, the cells were treated with free PTX, free VOR, PTX:VOR (PV), APVNs, L-APVNs, and Tf-L-APVNs at various concentrations (0.001 – 10 μM) and incubated for 24 h. After 24 h, cell viability was assessed by MTT assay (Sigma–Aldrich). MTT reagent (100 μL; 1.25 mg/mL) was added to each well and incubated for 3 h at 37 °C in the dark. The cells were then lysed and formazan crystals were dissolved by the addition of 100 μL of DMSO. The absorbance was measured at 570 nm using a microplate reader (Multiskan EX, Thermo Scientific, Waltham, MA, USA), and the combination ratio was evaluated using *Calcusyn* software (Biosoft, Cambridge, UK).

### Cellular uptake analysis

Briefly, cells (3 × 10^5^) from each cancer cell line were seeded in a 6-well plate and incubated for 24 h. The cells were then incubated with rhodamine-B loaded L-APVN and Alexa488-labelled Tf-L-APVN for various time intervals (0.5, 1, and 3 h) at 37°C. Cells were washed twice with PBS, detached using 0.25% trypsin/EDTA, centrifuged twice at 1500 rpm for 3 min, and the pellet was resuspended in 1 mL of PBS. The suspended cells were directly introduced into a FACS (fluorescence activated cell sorting (flow cytometer)) from BD FACS Calibur TM (USA) and analyzed with FL 1 and FL 2 channels. For confocal imaging, Alexa488 labeled Tf-L-APVNs were incubated in cancer cells for 1 h, washed two times with ice-cold PBS, fixed with 4% paraformaldehyde (10 min), and washed twice with PBS. The cells were pre-treated with excess free Tf 30 min before treatment to observe the competitive uptake of the Tf-L-APVNs into the cell. Finally, coverslips that contained cells were mounted onto slides and observed using confocal laser scanning microscopy (CLSM, Nikon A1+, Nikon, Tokyo, Japan).

### Annexin V/PI-based apoptosis assay and cell cycle analysis

The cells were seeded at a density of 2 × 10^5^ cells in a 6-well plate and incubated for 24 h. The cells were exposed to free PTX, VOR, PV, APVNs, L-APVNs and Tf-L-APVNs (1 μg/ml) for 24 h. The cells were stained with annexin V-FITC (BD Biosciences) and PI for 15 min and counted via flow cytometry using a FACS Calibur instrument (BD Biosciences). For cell cycle analysis, cells were treated with formulations (0.1 μM/mL) and incubated for 24 h. Afterwards, the cells were collected by trypsinization, centrifuged at 3000 rpm for 3 min at 4°C, washed twice with ice-cold PBS, fixed with 70% ice-cold ethanol, and incubated on ice for 1 h. The cells were then treated with 5 μL ribonuclease solution (10 mg/mL) and stained with 10 μL propidium iodide (1 mg/mL) for 30 min at 37°C in the dark. The DNA content of cells was measured by flow cytometry and the percentage of cells in each cell cycle phase was evaluated using CellQuest software (BD Biosciences).

### Cell migration assay

The scratch assay was performed to assess the effect of free PTX, VOR, PV, APVNs, L-APVNs, and Tf-L-APVNs on cancer cell migration. Cells (1 × 10^4^) were seeded in a 96-well plate and grown for 24 h. The cells were treated with respective drug formulations (0.1μg/ mL) for another 24 h. Cell monolayers were physically wounded by scratching the surface with a wound maker. Images of cells invading the scratch were captured after 16 h using the IncuCyte ZOOM™ (Ann Arbor, Michigan, USA). The pictures were evaluated by measuring the width of the wound with the IncuCyte ZOOM™ analysis software and the migration rate was expressed as a percentage of the scratch closure as follows:% of scratch closure = ((a – b)/a) × 100, where a is a distance between edges of the wound and b is the distance that remained cell-free during would closure.

### Immunoblot analysis

The cells were seeded at a density of 3 × 10^5^ and treated with respective formulations. The cells were then lysed on ice for 60 min in RIPA buffer (Thermo Scientific, MA, USA). The whole-cell lysates were subjected to sodium dodecyl sulfate-polyacrylamide gel electrophoresis (SDS-PAGE) and blotted onto a polyvinylidene difluoride membrane (Millipore, Billerica, MA). Membranes were blocked with 5% skim milk, and subsequently incubated with the specific primary antibodies (1:1000 dilution) overnight at 4°C. After incubation with secondary antibodies, proteins were detected using chemiluminesecence detection reagents. Films were developed using a Kodak imaging film (Kodak, USA) processor.

### Pharmacokinetic study

Male Sprague Dawley rats weighing 200 ± 10 g were divided equally into six groups with four rats in each group. The experimental protocols were in accordance with the guidelines framed by Institutional Animal Ethical Committee, Yeungnam University, South Korea. The right femoral artery was surgically cannulated to obtain blood samples while the left femoral vein was cannulated to administer the intravenous injection. The injection volume of different formulations was 0.3 mL (5 mg/kg) per animal. Blood samples (0.15 mL) were withdrawn at predetermined intervals (0.25, 0.5, 1, 2, 4, 6, 8, 10, 12, and 24 h) and immediately centrifuged (Eppendorf, Hauppauge, NY, USA) at 13000 rpm for 10 min. The plasma supernatants were stored at –20 °C until further analysis. The samples for HPLC analysis were prepared as following; 150 μl of thawed plasma was mixed with 150 μl of methanol and vortex-mixed for 30 min. The mixture was centrifuged, supernatant was evaporated, the samples were reconstituted in the mobile phase and 20 μl was injected into the HPLC system. The HPLC conditions were as discussed in the *in vitro* studies section. The plasma concentration–time values were modeled by non-compartmental analysis using WinNonlin software (professional edition, version 2.1; Pharsight Corporation, Mountain View, CA, USA). The pharmacokinetic parameters analyzed included the elimination rate (K_el_), half-life (t_1/2_), maximum plasma concentration (C_max_), time of C_max_ (T_max_), mean retention time (MRT), and area under the plasma concentration–time curve (AUC).

### *In vivo* antitumor study

HepG2 cell-bearing xenograft mice models were developed by subcutaneously injecting 1 × 10^7^ cells/100 μl into the rear flanks of 5-week-old male BALB/c nude mice (Orient Ltd. Osan, South Korea). After two weeks of initial implantation, animals were randomly divided into six groups (*n* = 6 per group). Respective formulations were injected at a dose of 100 μg/100 μL in 750 μg carrier by tail vein four times every 3 days. Tumors were measured twice weekly using a digital caliper and the tumor volume (V) was calculated as, V = ((width)^2^ x length)/2. The animals were sacrificed after four weeks of treatment and the primary tumor was excised, weighed, and subjected to further analysis.

### Histopathological analysis

Xenografted tumor masses were cut and fixed in 10% neutral buffered formalin, embedded in paraffin, serially sectioned (3–4 μm), and stained with H&E prior to histopathological examinations under a light microscope (Nikon, Tokyo, Japan). The tumor cell volumes and intact tumor cell-occupied regions (%/mm^2^ of tumor mass) were calculated using a computer-based automated image analyzer (iSolution FL ver 9.1, IMT i-solution Inc., Quebec, Canada).

### Immunohistochemical analysis

The apoptotic markers, caspase-3, PARP, CD31, and Ki67 were investigated immunohistochemically using purified primary antibodies and biotinylated secondary antibodies, with avidin-biotin-peroxidase complex (ABC) and a peroxidase substrate kit (Vector Labs, Burlingame, CA, USA). Briefly, endogenous peroxidase activity was blocked with methanol and 0.3% H_2_O_2_ for 30 min, and non-specific immunoglobulin binding was blocked with normal horse serum blocking solution in 10 mM citrate buffer (pH 6.0). Tissue sections were incubated with primary antisera overnight at 4°C in a humidity chamber, and then incubated with biotinylated universal secondary antibodies and ABC reagents for 1 h at room temperature. Finally, sections were reacted with the peroxidase substrate kit for 3 min at room temperature. Cells were counted as immunopositive if they had > 20% immunoreactivity to the apoptotic marker (caspase-3 or PARP). The percentage region occupied by caspase-3- and PARP-positive cells within the tumor mass (%/mm^2^ of tumor mass) was measured by an automated image analyzer.

### Statistical analysis

The results were analyzed with a one-way analysis of variance (ANOVA) test. Values were reported as mean ± standard deviation (SD) and the data were considered statistically significant at *p* < 0.05.

## SUPPLEMENTARY MATERIALS FIGURES AND TABLES


