# Supplementary material for: Molecularly targeted co-delivery of a histone deacetylase inhibitor and paclitaxel by lipid-protein hybrid nanoparticles for synergistic combinational chemotherapy
Source: Oncotarget. 2017 Jan 19;8(9):14925–40. doi: 10.18632/oncotarget.14742 (PMC5362455; doi:10.18632/oncotarget.14742)
Supplement: Supplementary file 1 [file oncotarget-08-14925-s001.pdf]

# Molecularly targeted co-delivery of a histone deacetylase inhibitor and paclitaxel by lipid-protein hybrid nanoparticles for synergistic combinational chemotherapy

## Supplementary Materials

## MATERIALS AND METHODS

### Physical state analysis

A differential scanning calorimeter (DSC-Q200, TA Instruments, New Castle, DE, USA) was used to study the thermal behavior of the samples. Differential scanning calorimetry (DSC) scans were recorded at a heating rate of 10°C/min from 40°C to 250°C. Fourier Transform Infrared Spectroscopy (FTIR) analysis was performed using a Thermo Scientific Nicolet Nexus 670 FTIR spectrometer over the range of 550−4000 $cm^{-1}$.

### Hemolytic analysis

Hemolytic toxicity was determined in a male Sprague-Dawley rat after treatment with different formulations. The experimental protocol was approved by the Institutional Animal Ethical Committee, Yeungnam University, South Korea. Briefly, the rat femoral artery was cannulated and whole blood samples were withdrawn. The blood was centrifuged, and red blood cells (RBC) were isolated and resuspended in saline. RBC suspension (2 mL) was dispersed in either saline solution or water to provide positive and negative controls, respectively. Test formulations (1 mL) were added to 2 mL of RBC suspension in separate tubes and adjusted to a final volume of 10 mL with normal saline. These suspensions were incubated at 37 ± 1°C for 30 min, followed by centrifugation at 3500 rpm for 10 min. The degree of hemolysis was determined by measuring the absorbance of the supernatant at 540 nm.

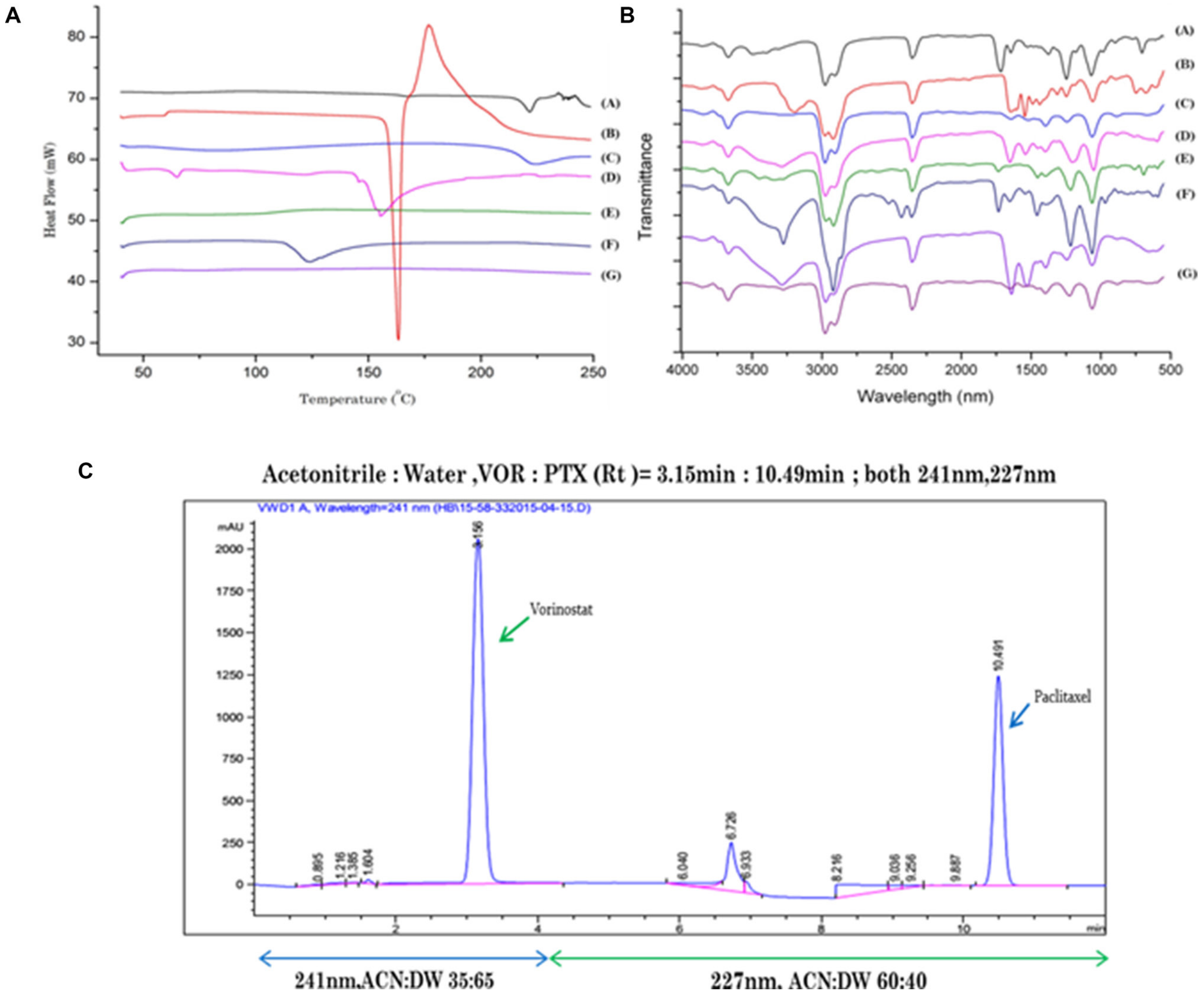


**Supplementary Figure 1:** (**A**) DSC and (**B**) FTIR analysis of free PTX (a), VOR (b), BSA (c), blank carrier (d), APVN (e), L-APVN (f), and Tf-L-APVN (g). (**C**) HPLC peaks of PTX ($R_t$: 10.49) and VOR ($R_t$: 3.15) in same base line by using gradient mobile phase.

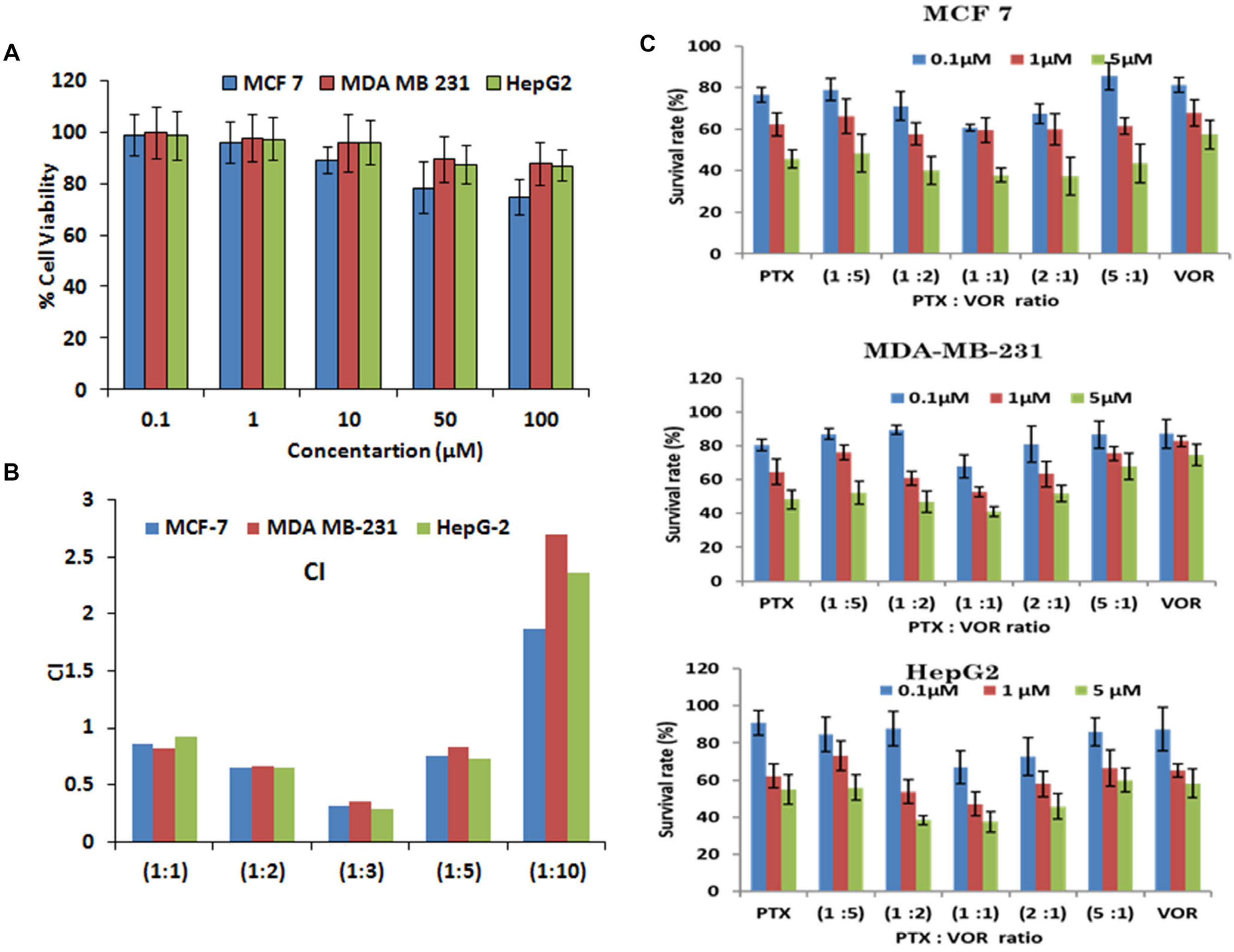


**Supplementary Figure 2:** (**A**) *In vitro* cell viability of blank carriers in a dose-dependent manner in MCF-7, MDA-MB-231 and HepG-2 cells at 24 h. (**B**) Combinational index and Synergism analysis of binary combinations of PTX and VOR in a ratiometric manner in (**C**) MCF-7, MDA-MB-231, and HepG2 cancer cells. The cells were treated with various PTX:VOR weight ratios 5:1, 2:1, 1:1, 1:2, and 1:5 at concentrations of 0.01, 0.1, and 1 µg/ml. Data are expressed as the mean ± SD (*n* = 6).

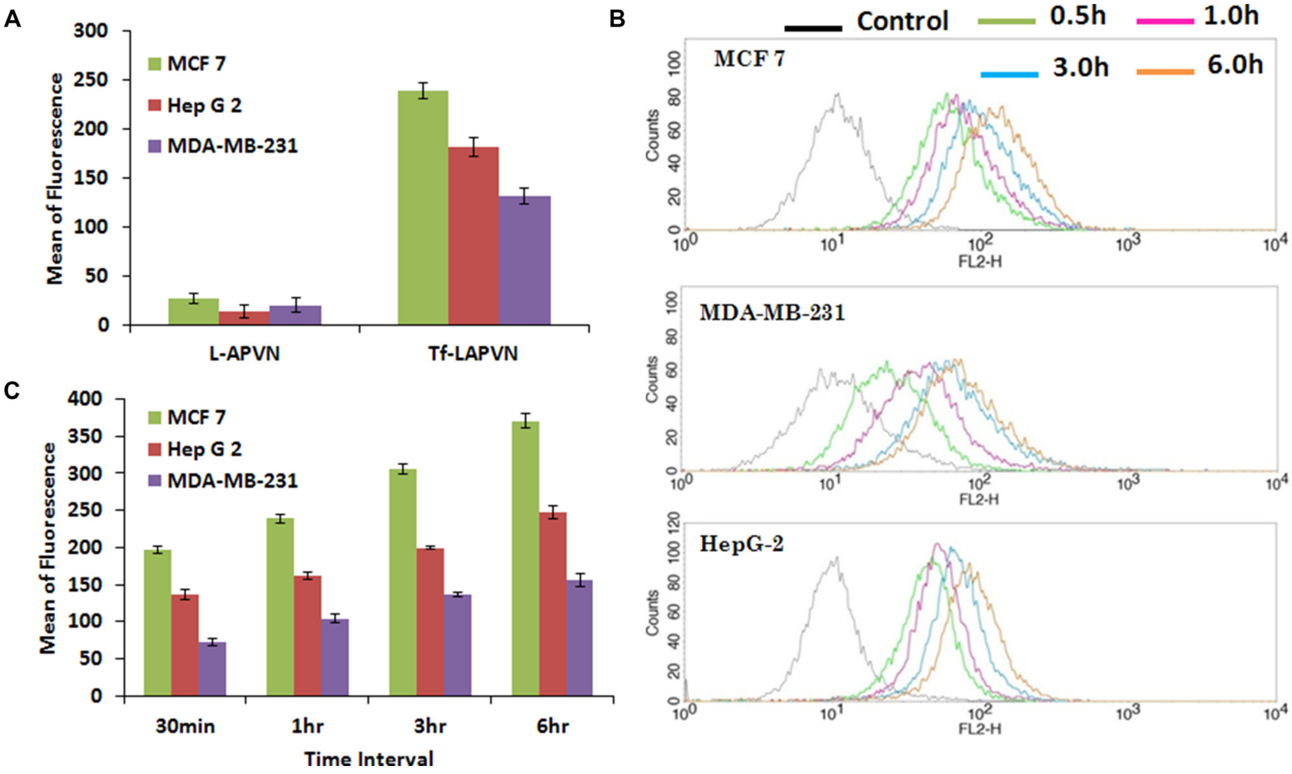


**Supplementary Figure 3:** (**A**) Quantitative mean fluorescence intensity of L-APVN and Tf-L-APVN in the indicated cancer cell lines. (**B**) Flow cytometry analysis of the cellular uptake efficiency of Tf-L-APVN in MCF-7, MDA-MB-231 and HepG2 cells incubated for 0.5, 1, 3, and 6 h. (**C**) Quantitative mean fluorescence intensity of Tf-L-APVN in the indicated cancer cell lines.

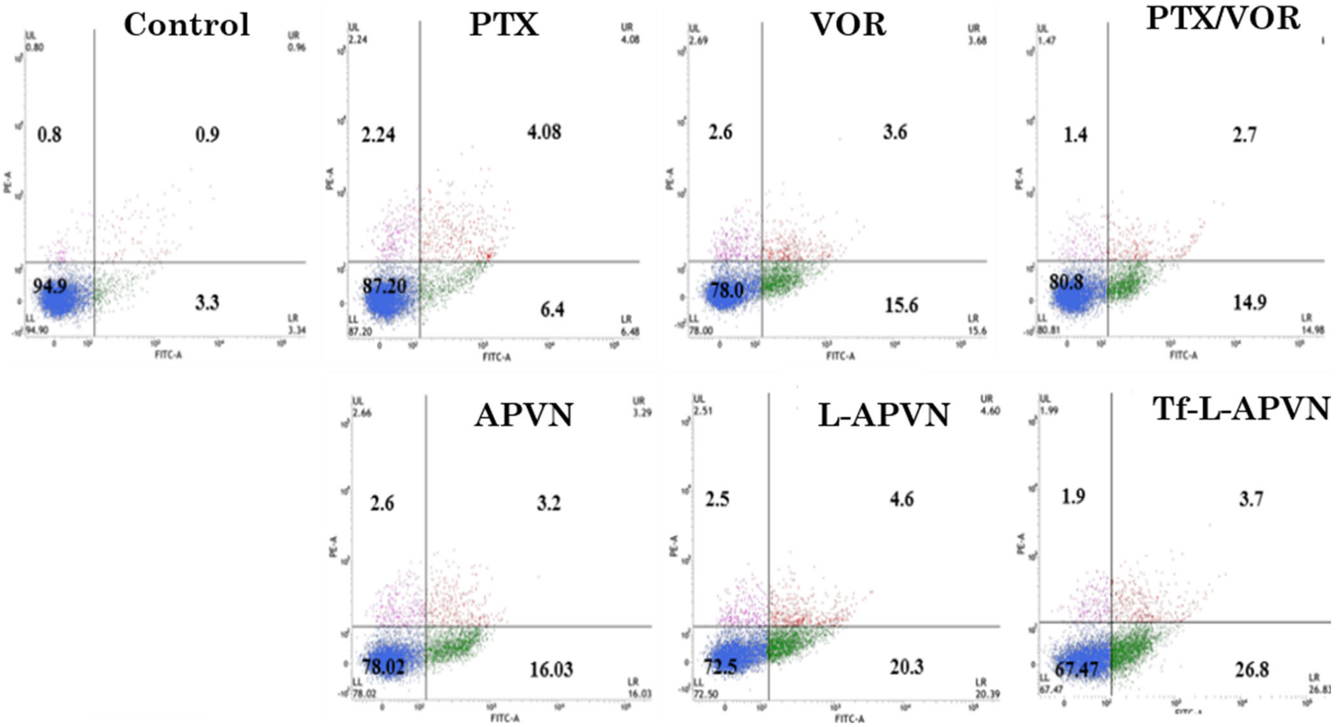


**Supplementary Figure 4:** Apoptosis induces by free drugs and formulations in MDA-MB-231 cell line.

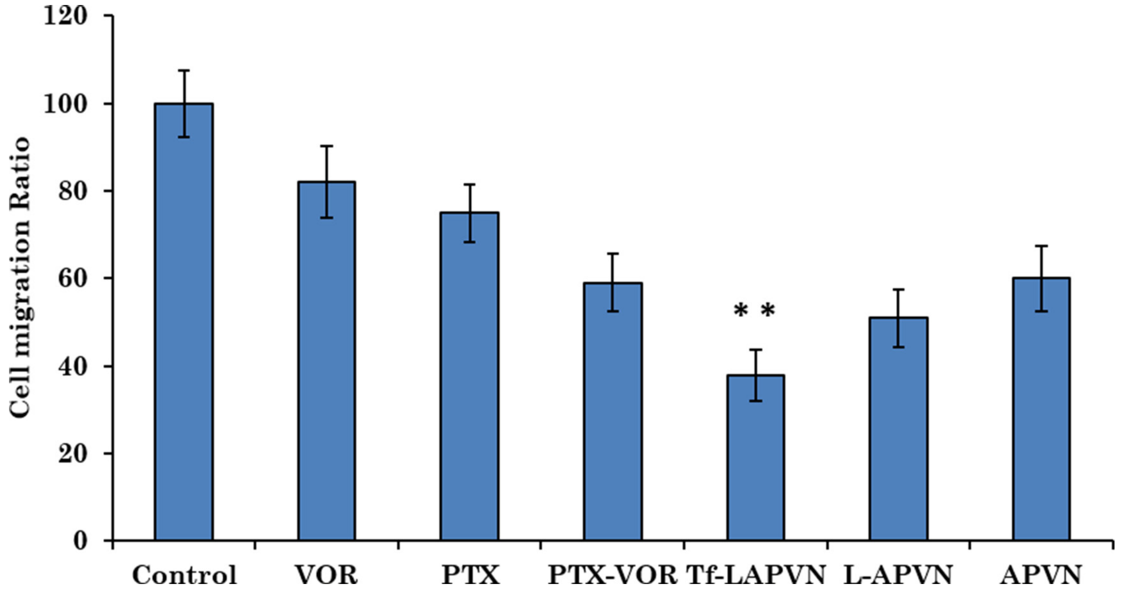


**Supplementary Figure 5:** Quantitative cell invasion ratio of different formulations at 0.1 μg/mL after 16 h of incubation in MCF-7 cells (*n* = 6).

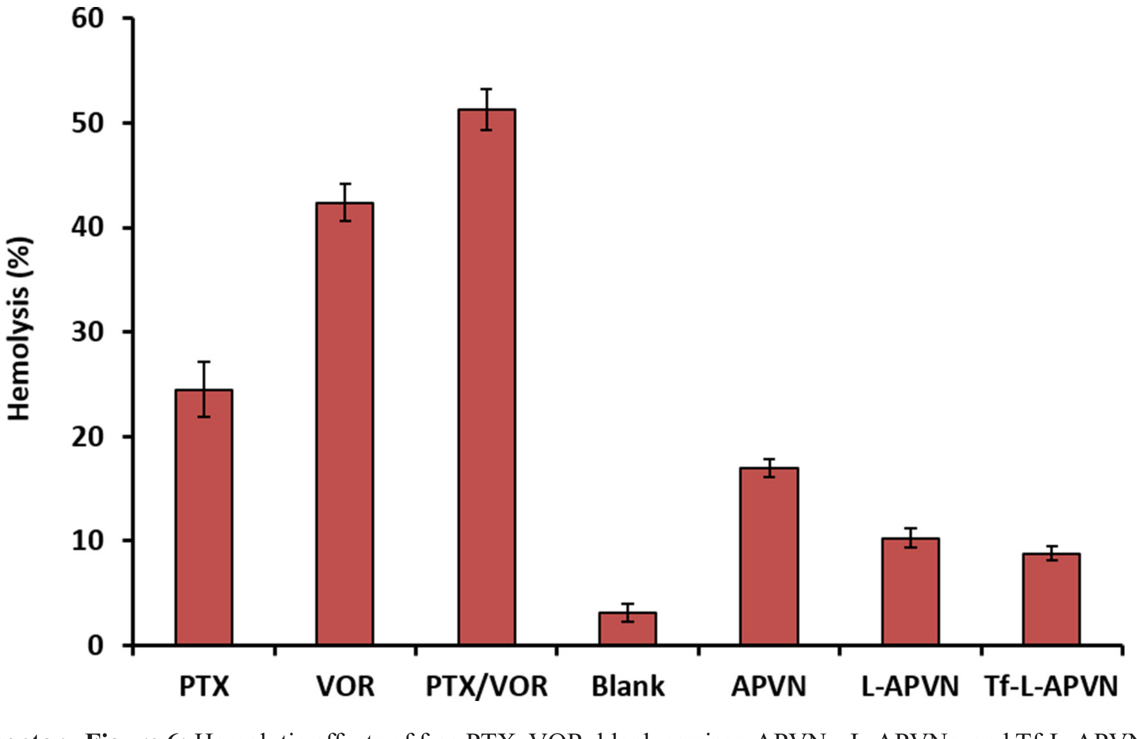


**Supplementary Figure 6:** Hemolytic effects of free PTX, VOR, blank carriers, APVNs, L-APVNs, and Tf-L-APVNs at 100 μg/mL. Each value represents the mean ± SD (*n* = 3).

**Supplementary Table 1: DLS characterization of APVN, L-APVN and $T_f$-L-APVN**

| Formulation | Particle size (nm) | PDI | ZP (mV) | EE (%) |
|---|---|---|---|---|
| APVN | 132.6 ± 2.4 | 0.157 ± 0.04 | –8.56 ± 3.7 | 98.7 ± 1.6 (PTX)<br>51.0 ± 4.8 (VOR) |
| L-APVN | 195.3 ± 3.6 | 0.224 ± 0.02 | –14.7 ± 2.8 | 100.0 ± 0.1 (PTX)<br>84.6 ± 1.3 (VOR) |
| Tf L-APVN | 232.1 ± 3.2 | 0.203 ± 0.01 | –16.2 ± 3.2 | 97.0 ± 0.5 (PTX)<br>77.2 ± 2.1 (VOR) |

Data expressed in mean ± SD (*n* = 3).

**Supplementary Table 2: Histomorphometric analysis of tumor masses form Xenograft bearing BALB/c nude mice**

| Groups | Tumor cell volumes (%/mm² of tumor mass) | Immunolabeled cell percentages (%/mm² of tumor mass) | | | |
|---|---|---|---|---|---|
| | | Caspase-3 | PARP | Ki-67 | CD31 (PECAM-1) |
| Control | 79.07 ± 10.04 | 4.26 ± 2.02 | 19.56 ± 4.79 | 59.37 ± 6.71 | 40.10 ± 4.68 |
| PTX | 53.84 ± 3.18[g] | 30.34 ± 5.00[a] | 29.59 ± 3.04[g] | 44.90 ± 5.31[g] | 30.54 ± 4.09[a] |
| VOR | 60.51 ± 10.92[g] | 26.31 ± 7.48[a] | 27.64 ± 3.22[h] | 42.64 ± 4.65[g] | 29.70 ± 2.52[a] |
| PTX/VOR | 47.12 ± 6.25[gk] | 48.16 ± 5.36[abc] | 43.78 ± 4.00[gij] | 23.07 ± 4.41[gij] | 21.62 ± 3.61[abc] |
| APVN | 40.53 ± 4.00[gij] | 43.93 ± 6.77[abc] | 47.12 ± 4.62[gij] | 21.81 ± 2.58[gij] | 19.46 ± 3.45[abc] |
| L-APVN | 38.48 ± 6.28[gij] | 58.52 ± 6.42[abc] | 47.42 ± 5.94[gij] | 20.67 ± 2.92[gij] | 21.71 ± 5.64[abc] |
| $T_f$-L-APVN | 29.80 ± 2.87[gijlmn] | 64.54 ± 5.61[abcdef] | 60.78 ± 8.18[gijlo] | 11.35 ± 2.54[gijlmn] | 8.12 ± 2.09[abcdef] |

Values are expressed as mean ± SD of six tumor mass histological fields
PARP = Cleaved poly(ADP-ribose) polymerase; PECAM-1 = Platelet endothelial cell adhesion molecule 1 (CD31)
MW test = Mann-Whitney U (MW) test
LSD test = least-significant differences multi-comparison (LSD) test

[a] $p < 0.01$ as compared with vehicle control by LSD test
[b] $p < 0.01$ as compared with F1 by LSD test
[c] $p < 0.01$ as compared with F2 by LSD test
[d] $p < 0.01$ as compared with F3 by LSD test
[e] $p < 0.01$ as compared with F4 by LSD test
[f] $p < 0.01$ as compared with F5 by LSD test

[g] $p < 0.01$ and [h] $p < 0.05$ as compared with vehicle control by MW test
[i] $p < 0.01$ as compared with F1 by MW test
[j] $p < 0.01$ and [k] $p < 0.05$ as compared with F2 by MW test
[l] $p < 0.01$ as compared with F3 by MW test
[m] $p < 0.01$ as compared with F4 by MW test
[n] $p < 0.01$ and [o] $p < 0.05$ as compared with F5 by MW test
